# Adult Attachment and Personal, Social, and Symptomatic Recovery From Psychosis: Systematic Review and Meta-Analysis

**DOI:** 10.3389/fpsyt.2021.641642

**Published:** 2021-02-24

**Authors:** E. M. M. van Bussel, N. H. M. Nguyen, A. I. Wierdsma, B. C. van Aken, I. E. M. G. Willems, C. L. Mulder

**Affiliations:** ^1^Geestelijke gezondheidszorg Oost Brabant, Institute for Mental Health, Oss, Netherlands; ^2^Department of Psychiatry, Erasmus Medical Centre, Epidemiological and Social Psychiatric Research Institute, Rotterdam, Netherlands; ^3^GGZ Breburg, Institute for Mental Health, Tilburg, Netherlands; ^4^Parnassia Psychiartric Institute, Rotterdam, Netherlands

**Keywords:** attachment, personal recovery, social recovery, symptomatic recovery, first episode of psychosis, schizophrenia

## Abstract

Despite growing evidence for the role of attachment in psychosis, no quantitative review has yet been published on the relationship in this population between insecure attachment and recovery in a broad sense. We therefore used meta-analytic techniques to systematically appraise studies on the relationship between attachment and symptomatic, social and personal recovery in clients with a psychotic disorder. Using the keywords attachment, psychosis, recovery and related terms, we searched six databases: Embase, Medline Epub (OVID), Psycinfo (OVID), Cochrane Central (trials), Web of Science, and Google Scholar. This yielded 28 studies assessing the associations between adult attachment and recovery outcome in populations with a psychotic disorder. The findings indicated that insecure anxious and avoidant attachment are both associated with less symptomatic recovery (positive and general symptoms), and worse social and personal recovery outcomes in individuals diagnosed with a psychotic disorder. The associations were stronger for social and personal recovery than for symptomatic recovery. Attachment style is a clinically relevant construct in relation to the development and course of psychosis and recovery from it. Greater attention to the relationship between attachment and the broad scope of recovery (symptomatic, social, and personal) will improve our understanding of the illness and efficacy of treatment for this population.

## Introduction

The process of recovery is a real challenge for people who have a psychotic disorder. Many of them not only have to overcome the symptoms of the disorder, but also have to deal with problems related to social functioning, including housing, work or education, social relationships, stigma, and identity. Despite the various evidence-based therapies for treating it (1, 2), it is still a very heterogeneous disorder, whose prognosis differs greatly between clients (3). If we are to improve our understanding of the illness and to improve treatment efficacy, we need to know why some clients with a psychosis can recover faster, more fully, and with fewer relapses than others.

Psychosis is an epigenetic disorder, whose etiology certainly includes biological factors, and whose known risk factors include interpersonal experiences such as early trauma and neglect (4, 5). Greater recognition of the impact of interpersonal experiences and stress has increased interest in Bowlby's attachment theory, which discusses the impact of early interpersonal relationships on stress regulation and functioning later in life (6, 7). As negative interpersonal experiences may increase a person's vulnerability to psychosis, it is possible that positive interpersonal experiences not only play a protective role in the development of psychotic symptoms, but also contribute to better recovery (8). A secure attachment style is considered to be associated with greater resilience, emotional well-being and mental health, and also with greater emotion regulation, hope, and optimism in life (9). Because recovery and attachment are complex, multidimensional concepts, the role of attachment in recovery from a psychotic disorder is a challenging field of research. It is nonetheless of great clinical importance, as it contributes to a better understanding of the course of the illness and efficacy of treatment of the psychotic disorder (9, 10). As a mediator between attachment and psychosis, mentalization is an aid to developing interventions that focus on helping clients to repair their understanding of their own mental states and those of others (11).

### Recovery

Recovery in schizophrenia and Serious Mental Illness (SMI) is a multidimensional concept that has evolved over time (12, 13). Although the main objective of mental health care, for many years, was symptomatic recovery—the reduction of symptoms and the improvement of physical functioning (14)—pressure from consumer-based groups caused attention to shift from a mainly clinical symptomatic perspective toward one that was more personal subjective (12, 15–17). Eventually this led to a conceptual framework for personal recovery in mental health known as the CHIME framework, an acronym representing 5 processes of personal recovery namely: Connectedness, Hope and optimism about the future, Identity, Meaning in life, and Empowerment (18). Personal recovery is a unique individual process in which a client gives meaning to previous events, and takes steps to regain a grip on their life (18).

There is also a third dimension of recovery, one that is both important and widely used. This is the process of social recovery, which includes the following aims: degrading the public stigma of mental illness and to improving not only the position and rights of clients and former clients within society (14), but also their housing, work, education and social relationships (19).

The three recovery dimensions can be viewed as an interactive model in which one dimension may influence the others. Symptomatic recovery is no longer treated as a prerequisite for social or personal recovery (2).

### Attachment

Attachment theory is a life span theory, proposing that children develop internal working models of the self and others through early relationships with caregivers. These working models are carried forward into adulthood (7), affecting the development not only of current and future stress regulation, but also of interpersonal functioning and relationships.

Attachment can be approached in two ways: categorical and dimensional. The categorical approach usually defines four main categories of attachment style: (20–23). The first, the secure (or autonomous) attachment style is thought to result from emotionally available and responsive primary caregivers, who allow the infant to explore in their presence, and who are also comfortable with shows of the child's emotional distress. This results in being comfortably with both intimacy and autonomy in adulthood. The second is the anxious attachment style (also referred to as “preoccupied” or “insecure ambivalent”). This is thought to result from a caregiver whose inconsistent availability led the infant to exaggerate emotional expression and minimize exploration of the environment, all in an attempt to maintain the caregiver's attention. In adulthood this is represented by heightened emotional expression and fear of autonomy and separation. The third is the avoidant attachment style (also referred to as “dismissing” or “insecure avoidant”). This is thought to develop from experiences of rejection by caregivers. In adulthood this can result in downplaying emotions and fear of intimacy. The fourth is the disorganized attachment style (also referred to as fearful) and is thought to arise as a response either to disrupted care experiences, such as neglect and early losses, or to frightening caregiver behavior such as physical and sexual abuse in childhood. These experiences lead a child to respond to their caregiver with fear or contradictory behaviors (21, 22). In adulthood, disorganized attachment is represented by contradictive behavior and an inconsistent sense of self. Although the categorical approach is often used in clinical practice, its disadvantage is that, in reality, clients rarely fit neatly into a single category. This problem can be bypassed by the use of two-dimensional instruments that measure the degree of avoidance and anxiety that people experience. Conceptually, the two-dimensional model is the underlying construct for the categorical approach. Clients who have a secure attachment style score low on anxiety and avoidance, those with an anxious attachment style score high on anxiety and low on avoidance, those who have an avoidant attachment style score high on avoidance and low on anxiety, and those with a disorganized attachment style, score high on both dimensions.

### Attachment and Recovery Related Outcome

Over the last 15 years, the importance of the different attachment styles in clients with psychosis has attracted more interest in the research field. A meta-analysis of the relationship between attachment and psychosis showed that the prevalence of insecure attachment styles was higher in individuals with psychosis (76%) than in non-clinical samples (38%). Especially the disorganized attachment style was the most prevalent (24). Furthermore, a weak relationship was found between insecure attachment and the severity of positive symptoms (24). Four narrative reviews found attachment insecurity to be associated with the following: poorer outcomes in psychosis, earlier onset of illness, less adaptive recovery styles, poorer quality of life and both a poorer therapeutic alliance and poorer engagement with mental health services (11, 25–27). Individuals with avoidant attachment styles also tended to be hospitalized for longer than those with secure attachment styles (28). With regard to attachment and social recovery in the psychotic population, insecure attachment was found to be associated with poorer social and individual living skills and less appropriate community behavior, and with the severity of interpersonal difficulties (11, 29, 30).

While the literature has discussed the relationships between various recovery outcomes and the concept of attachment in clients with a psychotic disorder, no meta-analysis or systematic review has examined attachment in relation to all the different aspects of recovery. The purpose of this systematic review and meta-analysis was therefore to give an overview of the relationships between adolescent attachment styles and adult attachment styles and symptomatic, social, and personal recovery amongst individuals with a non-affective psychotic disorder.

## Materials and Methods

Our review protocol was accepted into the Prospero database under registration number CRD42018102529; see https://www.crd.york.ac.uk/PROSPERO/.

### Inclusion and Exclusion Criteria

Studies were included in the analyses if (1) participants had been diagnosed with a non-affective psychotic disorder; (2) used a measurement of attachment in adolescents or adults (both defined as being 16 years or older); (3) used a measurement of personal, social or symptomatic recovery; (4) the study design measured a quantitative relationship between attachment and the different dimensions of recovery, except from case reports and systematic reviews; (5) they were in English. Studies were excluded if they solely described qualitative data, if they were single-case studies, conference abstracts, book chapters, reviews, unpublished studies or dissertations; and if they did not assess adolescent attachment or adult attachment in relation to outcomes associated with recovery. To focus specifically on adolescent attachment and/or adult-attachment, we excluded articles on attachment-related concepts such as loneliness, empathy, social cognition, social functioning, theory of mind (TOM), metacognition, mentalization, intimacy, object relations and schemes, parental-bonding or parental attachment, unless these concepts were studied next to or in combination with adult attachment. We also excluded articles that focused on at risk-mental state for psychosis (ARMS), unless this concept was studied in combination with a diagnosed psychotic disorder. Measurements of quality of life (QoL) were also excluded. Although QoL overlaps with the concept of recovery, it is still discussed in the literature as a distinct concept (31, 32).

### Search Strategy

To find empirical studies that focused specifically on attachment and recovery in clients who had been diagnosed with a psychotic disorder, we searched the following six databases: Embase, Medline Epub (OVID), Psycinfo (OVID), Cochrane Central (trials), Web of Science, and Google Scholar, using the keywords Attachment, Psychosis, and Recovery and related terms. Duplicate records were removed after the initial search. Hand searches were carried out in relevant journals and reference lists, and search results were cross-referenced with existing reviews (11, 25–27) for any additional studies that may have been missed. Online titles and abstracts were reviewed.

### Recovery Outcomes

With regard to symptomatic recovery, we included all outcomes involving a broad spectrum of instruments measuring symptom severity. Per study, we then categorized symptomatic outcomes into positive, negative and general symptoms per study. With regard to social recovery, we included all measurements involving participation in society and everyday life, that is, maintaining social relationships as well as other activities in daily life that are relevant to education, employment, housing and hobbies. And with regard to personal recovery, we used outcome measurements that fitted the CHIME conceptual framework (18), such as measuring hope, self-esteem, self-stigma and satisfaction with life domains.

As our study included mainly cross-sectional data, we did not apply the time criterion for recovery/remission [at least 6 months (33)]. However, the cross-sectional data notwithstanding, we are of the opinion that all participants had gone through a process of illness and recovery in some way. Due to the nature of the process underlying all the data, we therefore believe that the term “recovery” is appropriate.

### Measurement of Adolescent and Adult Attachment Style

With regard to measurements of attachment, we chose to include dimensional instruments as well as categorical ones. The two-dimensional model was found to be valid for measuring adult attachment (34). As it is often used as the underlying construct for the categorical approaches and other dimensional multi-item scales, we decided to use these underlying two dimensions where possible. The Revised Adult Attachment Scale (RAAS), for example, has three scales: discomfort depending on others, discomfort with closeness and anxiety about being unloved. The first two scales represent the avoidance dimension, while the third scale fits the anxiety dimension. A factor analysis of all existing self-report measures suggests that the use of multi-item scales with the two underlying dimensions or subscales—“anxiety” and “avoidance”—are valid for investigating adult attachment (35). We included self-reports (9) and assessments by the clinician or researcher (such as the Adult Attachment Interview, the AAI) (36). The AAI has been found to be reliable and valid for measuring adult attachment (37).

### Computation of Effect Sizes and Statistical Analyses

Pearson's correlation coefficient (*r*) was chosen as effect size because most studies reported associations either as correlations or regression coefficients. All correlations or corrected beta coefficients (38) are expressed in terms of a higher score representing a more problematic outcome. For longitudinal or intervention studies we combined baseline coefficients, when reported, with the effects sizes of cross-sectional studies. Where studies reported on multiple measures of type of recovery, we calculated the within-study average effect size to avoid violating the meta-analysis assumption of independence.

Separate meta-analyses were conducted for anxious or avoidant attachment and the distinct dimensions of recovery. For each meta-analysis, the pooled effect size was calculated using inverse-variance-weighted Fisher's *Z*-values. To evaluate the overall effect size, we used random-effects estimation and calculated 95% confidence intervals. Coefficients of 0.10 were interpreted as small effects, 0.30 as medium effects, and 0.50 as large effects. The Cochrane *Q* test and the *I*^2^ statistic were used to summarize variability in effect sizes between studies. Publication bias was explored using funnel plots and the Egger regression asymmetry test. Meta-regression analyses were conducted to explore between-study differences related to study population (recurrent psychosis or first-episode psychosis); type of questionnaire (self-report or interviewer rated); and gender.

Study quality was independently assessed by three raters using the NIH Quality assessment tool for observational and cross-sectional studies (39). This tool covers topics such as study objectives, sample selection, and adequate reporting, on the basis of which the methodological quality of each study is rated as poor, fair or good. We found large variations in study quality, as the association in many studies between attachment and recovery was not the primary research question. One study was rated as good, ten studies as fair, and 17 studies as poor. This limited the opportunities for exploring the effect of study quality.

Sensitivity analyses focused on the consequences of excluding studies that did not report correlation coefficients, or reported only the level of statistical significance. We repeated our analysis, in which the correlations reported as statistically non-significant were recorded as zero. Point estimates and confidence intervals were not greatly affected. A conventional alpha level of 0.05 was used for tests of heterogeneity and publication bias. All statistical analyses were performed using the metaphor package (40) in R (41).

Details of meta- and sensitivity analysis and tests of heterogeneity are available on request from the first author.

## Results

### Literature Search

**Figure 1:**
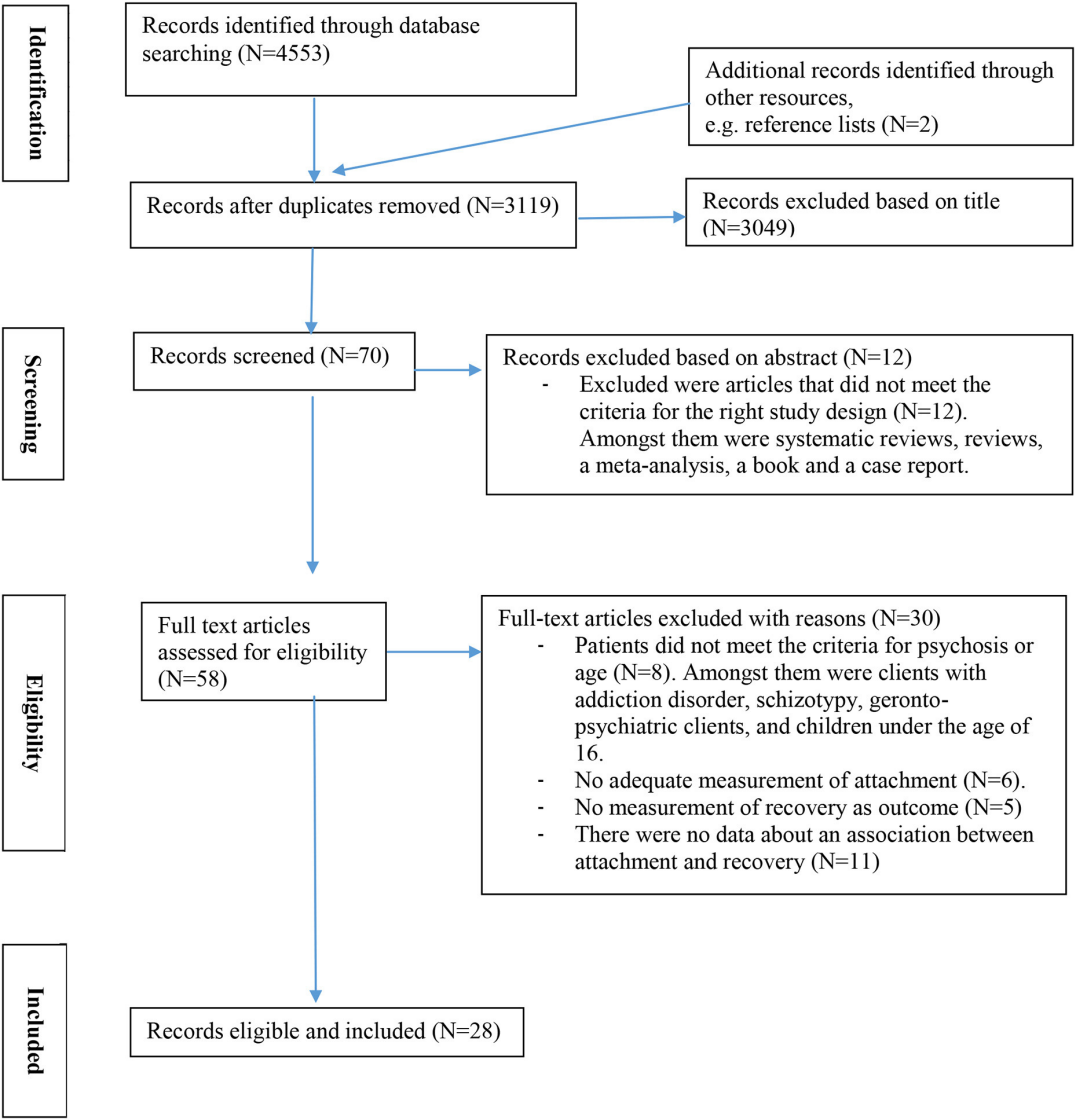
PRISMA flow diagram of systematic search.

The search and exclusion process is summarized in the PRISMA flow diagram of the systematic search (Figure 1). In the systematic process, the search was performed and the papers were rated independently by the first two authors before inclusion in the final sample.

### Study Participants Characteristics

The 28 studies relevant to our research question had been published between 2007 and 2020. Most were cross-sectional (*k* = 25). The participants' diagnoses met the criteria for first-episode psychosis in seven studies and for recurrent psychosis in 21 studies. In total, the studies included 2,598 participants with a psychotic disorder, 380 of whom had been diagnosed with a first episode of psychosis. The reported mean age ranged from 17 to 52.5 years; the participants' composite mean age was 36 years. However, no information on age was available in three studies (42–44). Although two studies included only men (28, 45), and although information on gender was not available in one study (46), 30.2% of overall participants were female.

Eight different measurements of attachment had been used (see Table 1), with three studies using narrative measurement (AAI) and 25 studies using self-report measurement. To conceptualize attachment, by far the highest number of studies used the dimensional approach. Most studies (*k* = 26) focused on symptomatic outcomes, but few referred to personal recovery (*k* = 4) or social recovery (*k* = 4). Data from 17 studies could be included in the meta analysis. Table 2 provides a summary of all the studies included.

**Table 1:** Instruments for measuring attachment.

**Measurement**	**Type**	**Scales and dimensions**	**Categories**	**Developers**
Adult attachment Interview (AAI)	Semi-structured interview	Q sort - Secure vs. insecure - Deactivating (avoidance) vs. hyper activating (anxiety)	CohT - Secure autonomous - Insecure dismissing - Insecure preoccupied - Unresolved	(47) (48)
Adult Attachment Scale (AAS and AAS-R and RAAS)	Multi item self-report	-Discomfort depending on others (avoidance) - Discomfort with closeness (avoidance) - Anxiety about being unloved (anxiety)		(49, 50)
Attachment Style Questionnaire (ASQ)	Multi item self-report	-Discomfort with closeness (Avoidance) - Relationships as secondary (avoidance) - Need for approval (anxiety) - Preoccupation with relationships (anxiety) - Confidence (anxiety)		(51)
Experiences in Close Relationships (ECR or ECR-R or ECR-RS)	Multi item self-report	-Anxiety - Avoidance		(35)
ESM attachment (experienced sampling method with six items from the AAS)	Multi item self-report	-Secure - Insecure		(46)
H and S, Hazan and Shaver's Adult Attachment Prototypes	Single item self-report		-Secure - Anxious - Avoidant	(52)
Psychosis attachment measure (PAM)	Multi item self-report	-Anxiety - Avoidance		(53)
Relationship questionnaire (RQ)	Single item self-report	-Anxiety - Avoidance	-Secure - Dismissive - Fearful - Preoccupied	(23)

**Table 2:** Overview of included studies.

**Nr**	**Study**	**Study design**	***N***	**Population**	**Mean age (SD)**	**Gender (%F)**	**Attach-ment mea surement**	**Attachment construct**	**Recovery outcome measurement**	**PS**	**NS**	**GS**	**PR**	**SR**	**Included in the meta-analysis**
1	(54)	CS	24	RP	32.9 (8.65)	37.5	PAM	DIM	CDSS PSYRATS	•		•			Yes
2	(55)	CS	35	RP	31.1 (7.86)	37.0	ECR-R	DIM	PANSS	•	•	•			Yes
3	(56)	CS	60	RP	40.2 (11.7)	63.3	RSQ	DIM	PC	•					Yes
4	(30)	L	96	RP	44.0 (12.8)	31.0	PAM	DIM	PANSS IIP-32	•	•	•		•	Yes
5	(57)	CS	73	RP	39.1 (11.3)	17.2	PAM	DIM	PANSS	•					Yes
6	(43)	CS	25	RP	NR	36.0	PAM	DIM	CORE-OM			•	•	•	Yes
7	(58)	CS	52	FEP	24.0 (12.33)	9.6	PAM	DIM	GAF					•	No
8	(59)	CS	588	RP	36.7 (12.33)	19.6	PAM	CAT	PSYRATS	•					No
9	(60)	CS	37	RP	37.1 (7.27)	19.0	ECR-RS	DIM	PC	•					Yes
10	(61)	CS	63	RP	40.4 (10.00)	30.0	ECR-R	CAT	PANSS	•					No
11	(29)	CS	96	FEP	23.7 (4.7)	34.0	ASQ	DIM	CASIG					•	Yes
12	(62)	CS	39	FEP	17.0 (1.21)	41.4	PAM	DIM	PANSS GPTS	•	•				Yes
13	(63)	CS	41	RP	52.5 (9.6)	36.6	AAS-R	DIM	PANSS	•	•				Yes
14	(64)	L	79	FEP	24.6 (7.08)	31.6	AAI (CohT)	DIM	PANSS (12 months)	•	•				No
15	(65)	CS	28	RP	41.6 (10.05)	29.0	AAI	CAT	BPRS			•			No
16	(66)	CS	32	FEP	17.1 (1.3)	40.0	PAM	DIM	GPTS	•					Yes
17	(67)	CS	500	RP	37.5 (11.7)	19.6	RQ	CAT/DIM	PANSS	•	•	•			Yes
18	(68)	CS	127	RP	44.6 (11.53)	34.0	PAM	DIM	PANSS CDSS BDI-II	•	•	•			Yes
19	(42)	CS	34	FEP	23.32 (7.59)	42.0	AAI	CAT	PANSS	•	•	•			No
20	(69)	CS	55	RP	42.16 (11.33)	20.0	PAM	DIM	PSYRATS	•					Yes
21	(28)	CS	30	RP	38.4 (10.2)	0.0	H and S	CAT/DIM	PANSS	•	•	•			No
22	(70)	CS	100	RP	40.3 (11.2)	30.0	RQ	CAT	PANSS	•					No
23	(45)	CS	52	RP	46.64 (9.15)	0.0	ECR	DIM	PANSS BHS RSES	•	•	•	•		Yes
24	(71)	CS	48	FEP	35.3 (8.71)	52.1	RQ	CAT	ISMI				•		No
25	(46)	L (6 days)	20	RP	41.05 (12.53)	NR	AAS (ESM)	DIM	PDS(ESM) Hallucination (ESM)	•					No
26	(44)	CS	47	RP	NR	36.3	RQ	CAT	SCL-90R			•			No
27	(72)	CS	50	RP	33.8 (12.0)	38.0	RAAS	DIM	PANSS	•	•				Yes
28	(73)	CS	176	RP	37.6 (11.8)	30.0	RQ	DIM	PANSS PaDs SERS	•			•		Yes

*Summary of included studies*.

*CS, cross-sectional; L, longitudinal; RP, recurrent psychoses; FEP, first episode psychosis; DIM, dimensional; CAT, categorical; PS, positive symptoms; NG, negative symptoms; GS, general symptoms; PR, personal recovery; SR, social recovery; SD, standard deviation; N, number of included clients*.

**Attachment measures**; AAI, Adult Attachment Interview; AAI (CohT), Adult Attachment Interview (Coherence of Transcript); AAS, Adult Attachment Scale; AAS-R, Adult Attachment Scale revised; AAS (ESM), experienced sampling method with items from the AAS; ASQ, Attachment Style Questionnaire; ECR, Experiences in Close Relationships Scale; ECR-R, Experiences in Close Relationships Scale Revised; ECR-RS, Relationship Structures questionnaire of the Experiences in Close Relationships-Revised; H and S, Hazan and Shaver's Adult Attachment Prototypes; PAM, Psychosis Attachment Measure; RAAS, Revised Adult Attachment Scale; RQ, Relationship Questionnaire.

***Recovery outcome measures;** BDI-II, Beck Depression Inventory-II; BPRS, Brief Psychiatric Rating Scale; BHS, Beck Hopelessness Scale; CDSS, Calgary Depression Scale for Schizophrenia; CASIG, Client Assessment of Strengths Interests and Goals; CORE-OM, Clinical Outcomes in Routine Evaluation—Outcome Measure; GAF, Global Assessment of Functioning; GPTS, Green Paranoid Thought Scale; IIP-32, Inventory of Interpersonal problems-32; Hallucination ESM (Experienced Sampling Method), hallucination items for ESM (experienced Sampling Method); Inventory of Interpersonal Problems-II; ISMI, Internalized Stigma of Mental Illness Inventory; PaDs, Persecution and Deservedness Scale; PC, Paranoia Checklist; PANNS, Positive and Negative Syndrome Scale; PDS (ESM), Persecution and Deservedness Scale items for ESM (Experienced Sampling Method); PSYRATS, Psychotic Symptom Rating Scale; RSES, Rosenberg Self-Esteem Scale; SCL-90R, Symptom Checklist-90-Revised; SERS, Self-Esteem Rating Scale*.

### Symptomatic Recovery

Positive symptoms had medium associations with the anxious attachment style (*r* = 0.24, 95% CI: 0.16–0.33, *k* = 15) and the avoidant attachment style (*r* = 0.20, 95% CI: 0.14–0.26, *k* = 15) (Tables 3 and 4). Although there was some variation in outcome due to high correlations in two first-episode-population (FEP) studies (62, 66), three FEP studies that were not included in the meta-analysis found no association between insecure attachment and positive symptoms (42, 58, 64).

**Table 3:** Anxious attachment and recovery outcome.

**Recovery**	***N***	**Mean ES**	***P***	**95% CI**	***I*^**2**^**	**Homogeneity (*Q*, df)**
**Symptomatic**						
Positive Symptoms	15	0.24	<0.001	0.16 to 0.33	40%	*Q* (df = 14) = 24.9, *p* = 0.035
Negative symptoms	8	0.02	0.477	−0.04 to 0.09	1%	*Q* (df = 7) = 6.83, *p* = 0.088
General symptoms	7	0.28	<0.001	0.17 to 0.37	35%	*Q* (df = 6) = 11.3, *p* = 0.079
**Social**	3	−0.47	0.0116	−0.72 to 0.11	86%	*Q* (df = 2) = 15.7, *p* = 0.000
**Personal**	3	−0.39	<0.001	−0.49 to 0.28	1%	*Q* (df = 2) = 3.37, *p* = 0.186

*N, number; mean ES, mean effect size; P, significance; CI, confidence interval; I^2^, I^2^ statistic; Q, Cochrane Q test*.

**Table 4:** Avoidant attachment and recovery outcome.

**Recovery**	***N***	**Mean ES**	***P***	**95% CI**	***I*^**2**^**	**Homogeneity (*Q*, df)**
**Symptomatic**						
Positive Symptoms	15	0.20	<0.001	0.14 to 0.26	9%	*Q* (df = 14) = 12.8, *p* = 0.540
Negative symptoms	8	0.09	0.0045	−0.03 to 0.16	1%	*Q* (df = 7) = 11.1, *p* = 0.133
General symptoms	7	0.20	<0.001	0.11 to 0.29	25%	*Q* (df = 6) = 6.45, *p* = 0.374
**Social**	3	−0.27	<0.001	−0.39 to 0.14	0%	*Q* (df = 2) = 1.59, *p* = 0.453
**Personal**	3	−0.31	<0.001	−0.42 to 0.20	0%	*Q* (df = 2) = 0.10, *p* = 0.952

*N, number; mean ES, mean effect size; P, significance; CI, confidence interval; I^2^, I^2^ statistic; Q, Cochrane Q test*.

Two other studies, that were not included in the meta-analysis, found positive associations only between attachment avoidance and positive symptoms (61, 70).

For negative symptoms, we found no association with anxious attachment (*r* = 0.02, 95% CI: −0.04 to 0.09, *k* = 8) and only a weak association with the avoidant attachment style (*r* = 0.09, 95% CI: 0.03 to 0.16, *k* = 8) (Tables 3 and 4). However, in one longitudinal FEP study (64), an association was found between insecure attachment styles (AAI) and negative symptomatology after 12 months.

Positive associations were found between the anxious attachment style and general symptoms (*r* = 0.27, 95% CI: 0.17–0.37, *k* = 7) (Table 3). As different questionnaires had been used to measure general symptomatology (Table 2), some variation in outcome was related to the type of questionnaire. The self-report questionnaires scored higher.

The association between general symptoms and the avoidant attachment style was also positive (*r* = 0.20, 95% CI: 0.11–0.29, *k* = 7) (Table 4). We found a mild variation in outcome and an indication of publication bias (funnel-plot asymmetry: *z* = 2.525, *p* = 0.012). Studies that were not included in the meta-analysis (*k* = 4) were rated as being of lower quality.

### Social Recovery

Four studies investigated the association between attachment and social recovery (29, 30, 43, 58). We included their use of the IIP-32, CORE-OM, CASIG, and GAF total as a measurement of social recovery outcome. Higher scores on anxious attachment (respectively, *r* = −0.47, 95% CI: −0.72 to −0.11, *k* = 3) and avoidant attachment (*r* = −0.27, 95% CI: −0.39 to 0.14, *k* = 3) (Tables 3 and 4) were related to a lower score on social recovery. In one FEP study that was not included in the meta-analysis, there was no evidence that attachment significantly predicted social outcome measured on the basis of total GAF scores (58).

### Personal Recovery

Four studies reported on the association between attachment styles and personal recovery measured with CORE-OM, SERS, RSES or ISMI (43, 45, 71, 73). Higher scores on anxious attachment (*r* = −0.39, 95% CI: −0.49 to −0.28, *k* = 3) and avoidant attachment (*r* = −0.31, 95% CI: −0.42 to −0.20, *k* = 3) (Tables 3 and 4) were related to lower scores on personal recovery. One FEP study that was not included in the meta-analysis had used the ISMI to measure the relationship between self-stigma and attachment style (71). Clients with anxious and avoidant attachment styles were more prone to self-stigma than those with a secure attachment style.

## Discussion

Overall, this study of the relationship between attachment and recovery found positive associations between both the anxious and avoidant attachment styles and psychotic and general symptoms. We found weak associations between both these attachment styles and negative symptoms. Higher scores on anxious attachment and avoidant attachment were also related to lower scores on social and personal recovery.

The positive associations we found between both these attachment styles and positive psychotic symptoms are in line with those in previous reviews (11, 25–27) and with a previous meta-analysis (24). In our meta-analysis and review, however, we found stronger positive associations in two FEP studies (62, 66) and no associations in three other FEP studies (42, 58, 64). These stronger positive associations may be related to the properties of the Green Paranoid Thought scale (GPTS). The GPTS measures both non-clinical and clinical paranoia, probably making this scale more sensitive than the PANNS for purposes of detecting paranoia in the FEP population. The absence of associations in the other FEP studies (42, 58, 64) can be attributed to the good symptomatic recovery in the first-episode group. The way attachment relates to psychotic symptoms can be understood by the fact that insecure attachment styles are accompanied by beliefs about self and others that fuel paranoid thinking (74). The relationship between attachment and voice hearing is more complex. While the attachment theory does not in itself offer an adequate account of the complexity of voice hearing, the onset of voice hearing commonly occurs during an important transition in attachment relationships. This suggests that there is a mutual relationship between them (74).

With regard to the negative symptoms, other reviews and the meta-analysis report equally inconsistent and weak findings in this field (11, 24–27). However, one longitudinal FEP study in our review, did find a positive association after 12 months (64). This variance in outcome may be explained by an earlier finding of a higher prevalence of negative symptoms in first-episode psychosis (50–90%) than in a population with schizophrenia (20–40%) (75). Some studies have attributed the lack of an association to a low score on negative symptoms in the research population (62, 68). It has also been argued that negative symptoms reflect a neurodevelopmental disorder, and are not therefore associated with attachment related-disruptions (76).

The positive associations found between the anxious and avoidant attachment styles and general symptoms support the idea that insecure attachment is a risk factor for psychopathological symptoms in general (9). Attachment insecurities contribute nonspecifically to many kinds of mental dysregulation because of their negative effects on central psychological resources: feelings such as optimism, hope and self-worth; and intra- and interpersonal regulatory skills (9). Although many insecurely attached people do not suffer from a mental disorder, attachment insecurity seems to amplify the impact of other pathogenic factors (9). In previous reviews, only Gumley et al. (26) addressed affective symptoms in relation to insecure attachment and found positive associations. In our review, two studies, including one FEP study, reported that the severity of general symptoms was not associated with a particular attachment style (28, 42).

With regard to social and personal recovery, higher scores on anxious attachment and avoidant attachment were related to lower scores on social and personal recovery. The associations found in both domains, were stronger than in the symptomatic recovery domain. In one FEP study, however, no evidence was found that insecure attachment significantly predicted social outcome measured by total GAF scores (58). As Berry states, although the relationship between attachment style and social functioning in psychosis is a potentially important area of research, it is seldom investigated (74). It is important particularly because outcomes in social recovery lag behind those in other recovery domains (77). Attachment and social functioning are related concepts. It has been shown that insecure attachment is characterized by negative views about acceptance, reassurance and safety in interpersonal relationships, and that the continued presence of an insecure internal working model of relationships can lead to poor social cognitive skills and result in social difficulties (25).

We found only limited operationalization's in the domain of personal recovery (self-esteem, hope, and self-stigma). As with social recovery, there is an overlap in the concepts of attachment and personal recovery as the inner representation about the self (or self-esteem) is a part of both concepts. Although we found associations between the anxious and avoidant attachment styles and personal recovery, too little research has been conducted to date to allow us to draw conclusions in this area.

### Limitations

The primary aims and research questions varied widely between the studies we included and did not necessarily focus on the relationship between attachment and recovery. Although the data extracted from these studies were relevant to our study, they were—from the point of view of the primary authors—sometimes an incidental finding. The research in the included studies was also relatively heterogeneous, with little methodological consistency or overlap. Attachment was measured in various ways using a variety of questionnaires. The issue of attachment is complicated by the number of terms or labels used to distinguish its different forms, with different names often being used for the same constructs.

With regard to the outcomes, we have discussed the terminology and the applicability of the term “recovery” and have concluded that recovery implies an improved state of functioning and wellbeing relative to an earlier state. Since attachment was assessed after the diagnosis of psychotic disorder, we also cannot rule out the effect of the psychotic disorder on the scoring of the attachment scales.

Finally, we found not only that very few studies had examined the domains of social and personal recovery, but also that the examination of social and personal recovery had not captured all possible aspects of the concept.

## Conclusions and Recommendations

Overall, the available evidence provides support for the role of attachment in the process of symptomatic, social and personal recovery. However, as a large majority of the studies were cross-sectional, and over half were rated as poor, the evidence needs to be interpreted with caution. In addition, the concepts of social and personal recovery are underexposed and research has failed to focus on all their aspects. Further research is necessary if we wish to generate greater understanding of prognosis and to improve treatment efficacy for clients who have undergone a psychotic episode. When we make the step to clinical practice, not only the process needed to achieve recovery is important but also the process necessary to maintaining it. If better long-term recovery is to be achieved, this will require greater insight and appropriate interventions, particularly regarding the process of recurrence and stagnation.

It is important that future research examines the domains of recovery as an entire, fully balanced concept. Given the interaction between the domains (symptomatic, social, and personal recovery), recovery in one domain can be supportive or protective of recovery in another domain. To provide greater insight into the ways in which attachment supports or hinders the process and long-term maintenance of recovery, there is also a need for longitudinal research.

The distinction between the first-episode population and the recurrent-episode population is important, as the two may differ with regard to the ways in which attachment affects prognosis. More data on how attachment influences recovery will support clients and professionals in improving diagnostics and treatment efficacy. Given that mentalization is seen as a mediator between attachment and psychosis, current therapies for psychosis and the recovery process may be improved by developing interventions that focus on helping clients to repair their understanding of their own mental states and those of others (11).

## Data Availability Statement

The original contributions presented in the study are included in the article/supplementary material, further inquiries can be directed to the corresponding author.

## Author Contributions

EB and NN have written this systematic review and meta-analysis, under the direct guidance of AW, BA, IW, and CM. All authors have commented on multiple versions of this manuscript. All authors have read and approved the final manuscript.

## Conflict of Interest

The authors declare that the research was conducted in the absence of any commercial or financial relationships that could be construed as a potential conflict of interest.
